# Surface plasmon resonance biosensing of the monomer and the linked dimer of the variants of protein G under mass transport limitation

**DOI:** 10.1016/j.dib.2016.10.029

**Published:** 2016-11-05

**Authors:** Hiroshi Imamura, Shinya Honda

**Affiliations:** Biomedical Research Institute, National Institute of Advanced Industrial Science and Technology, 1-1-1 Higashi, Tsukuba, Ibaraki 305-8566, Japan

## Abstract

This article presented the data related to the research article entitled “Calibration-free concentration analysis for an analyte prone to self-association” (H. Imamura, S. Honda, 2017) [1]. The data included surface plasmon resonance (SPR) responses of the variants of protein G with different masses under mass transport limitation. The friction factors of the proteins analyzed by an ultracentrifugation were recorded. Calculation of the SPR response of the proteins was also described.

**Specifications Table**

**Table:** 

Subject area	*Chemistry, Biology, Biochemistry*
More specific subject area	*Surface plasmon resonance (SPR) biosensing*
Type of data	*Figure, text*
How data was acquired	*Surface plasmon resonance data were collected on a Biacore T200 (GE Healthcare UK Ltd., England). Sedimentation velocity data were collected on a ProteomeLab XL-I (Beckman Coulter, Inc., Brea, CA).*
Data format	*Analyzed*
Experimental factors	*Binding of protein G variants with molecular weights of 6.4 kDa and 18.3 kDa to a ligand, immunoglobulin G1, and the sedimentation of the proteins were measured.*
Experimental features	*An experimental data in terms of supporting a theoretical SPR response, Measurement of diffusion-related parameters by an analytical ultracentrifugation.*
Data source location	*National Institute of Advanced Industrial Science and Technology, Tsukuba, Japan*
Data accessibility	*Data are provided within this article.*

**Value of the data**

• The present experimental SPR responses of proteins with different masses under mass transport limitation could be valuable for researchers interested in improvement or progress of a theory for calibration-free concentration analysis.
• Calculated data of a theoretical SPR responses help interpretation of experimental data.
• The friction factors of the monomer and the linked dimer determined by an analytical ultracentrifugation could be helpful data for researchers interested in diffusion of multimers.

## 1 Data

**Fig. 1:**
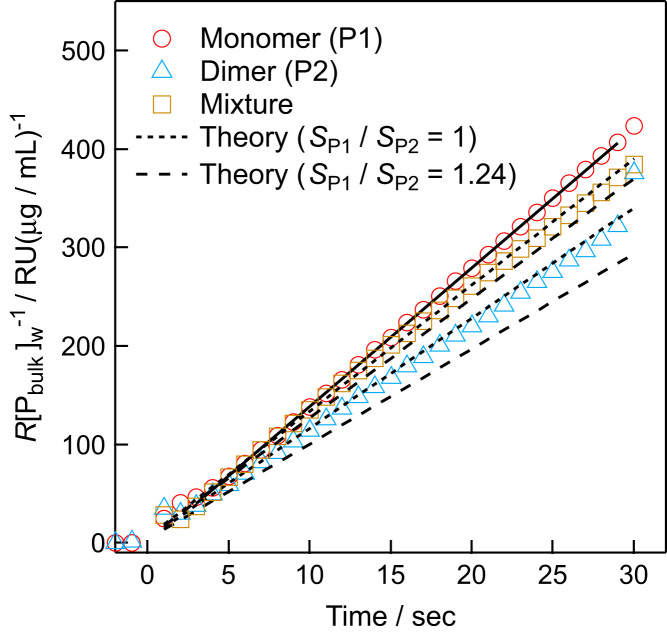
Sensorgrams of the analytes, the variants of Protein G, under a flow rate of 30 μL/min over a ligand surface consisting of immunoglobulin G1. The protein solutions of the monomer (red circles), the linked dimer (cyan triangle), and their mixture (dark orange square), the composition of which was *γ*=0.43, were analyzed. *R* is the response signal. [P_bulk_]_W_ represents the concentration of the analytes. A linear function was used to fit the data, indicated by the solid line. The theoretical slopes calculated by Eqs. (10), (11), (12), (13), (14) are indicated by the dotted lines (*s*_P1_/*s*_P2_=1) and the dashed lines (*s*_P1_/*s*_P2_=1.24 calculated by using the parameters, (*f*/*f*_0_)_P1_=1.25, (*f*/*f*_0_)_P2_=1.57, *v*_P1_=0.730 cm^3^/g, and *v*_P2_=0.716 cm^3^/g); the slope of (d*R*_P1_/d*t*) / [P1_bulk_]_W_, experimentally determined, was used as a reference to calculate the theoretical slopes of (d*R*_P2_/d*t*) / [P2_bulk_]_W_ and (d*R*/d*t*) / [P_bulk_]_W_. (For interpretation of the references to color in this figure legend, the reader is referred to the web version of this article).

In this data article, the data of the SPR measurements using a system with Protein G and Immunoglobulin G1 (IgG1) as an analyte and a ligand, respectively, are measured. The experimental data and the calculated curve according to equations described in Ref. [1] are presented (Fig. 1). The analytical centrifugation measurement of the analytes is documented.

## 2 Experimental design, materials and methods

### 2.1 Sample

The variant of streptococcal protein G B1 domain (Q32H/D36E/N37H/D40H/E42H/D47P/A48E), PG0919, designed and characterized previously [2], is a monomeric IgG-binding protein. A tandem protein, in which two PG0919 are connected by a linker sequence (unpublished), is regarded as the dimer of PG0919. PG0919 and the linked dimer of PG0919, both of which were expressed in *Escherichia coli* and purified as previously reported [2], were used. The lyophilized powder of the proteins was dissolved in water and dialyzed against HBS-T buffer solution composed of 0.01 M HEPES, 0.15 M NaCl, and 0.05% (v/v) polyoxyethylene (20) sorbitan monolaurate (pH 7.4). The ultraviolet absorption was measured by V-730BIO (JASCO Co. Ltd., Japan) using a quartz cuvette with a 1 cm path length to determine the protein concentration. The extinction coefficients at 280 nm, calculated based on the amino acid sequence [3], were 1.551 cm^−1^ (g/L)^−1^ for PG0919 and 1.628 cm^−1^ (g/L)^−1^ for the linked dimer of PG0919.

### 2.2 Surface plasmon resonance (SPR)

SPR measurement was performed with a Biacore T200 (GE Healthcare UK Ltd., England). 10 kRU (response unit; 1 RU=1 pg/mm^2^) of a monoclonal humanized immunoglobulin G1 with a molecular weight of 148 kDa was immobilized on a sensor chip CM5 (GE Healthcare UK Ltd.). HBS-T buffer solution was used for the measurement. The temperature was set at 298 K. The concentrations of PG0919 and the linked dimer of PG0919 were 9.28×10^−2^ and 7.08×10^−2^ µg/mL, respectively.

### 2.3 Analytical ultracentrifugation

Sedimentation velocity measurements were performed on a ProteomeLab XL-I (Beckman Coulter, Inc., Brea, CA) with rotor speeds of 40 krpm at 293 K. Absorbance at 280 nm was used to monitor the protein concentration in a double sector cell. Prior to the measurements, the proteins were dialyzed overnight against a buffer solution containing 0.01 M sodium phosphate and 0.15 M NaCl (pH 6.9). The initial protein concentrations were set at 0.6 and 0.5 mg/mL for PG0919 and the linked dimer of PG0919, respectively. The sedimentation velocity data were processed with the program SEDFIT using sedimentation coefficient distribution analysis [4].

### 2.4 Mathematical equations

The additional amino acid residues introduced into the linked dimer of PG0919 make the molecular weight (18,375 Da) 2.86 times larger than that of PG0919 (6427 Da), the monomer. Because the ratio between the molecular weights of the monomer and the linked dimer is non-natural number, the formulas for calculating a mass-dependent SPR response described in the reference [1] are tuned and written here, although the essence of the formulation is identical. When the analyte is a mixture of the monomer and the linked dimer, each of which has a one-to-one binding with a ligand, the SPR response signal, *R*, is expressed as:

(1) $$R=R_{P1}+R_{P2}$$

where the P1 and P2 subscripts designate the monomer and the linked dimer, respectively. The time dependence of *R* is given by:

(2) $$dR/dt=dR_{P1}/dt+dR_{P2}/dt,$$

where d*R*_P1_/d*t* = *M*_P1_*Gk*_c,P1_[P1_bulk_], d*R*_P2_/d*t* = *M*_P2_*Gk*_c,P2_[P2_bulk_], *M* is the molecular weight of the analyte, *G* is a factor converting concentration to an *R* value, and *k*_c_ is the mass transport coefficient. [P1_bulk_] and [P2_bulk_] are the concentration of the monomer and the linked dimer, respectively, in the bulk. The weight concentration of the monomer and the linked dimer are expressed by:

(3) $${\left[P1_{\mathrm{bulk}}\right]}_{W}=M_{P1}\left[P1_{\mathrm{bulk}}\right]$$

and,

(4) $${\left[P2_{\mathrm{bulk}}\right]}_{W}=M_{P2}\left[P2_{\mathrm{bulk}}\right].$$

Eq. (2) can be rewritten as:

(5) $$dR/dt=Gk_{c,P1}{\left[P1_{\mathrm{bulk}}\right]}_{W}+Gk_{c,P2}{\left[P2_{\mathrm{bulk}}\right]}_{W}.$$

The total weight concentration of the analytes, [P_bulk_]_W_, is defined by:

(6) $${\left[P_{\mathrm{bulk}}\right]}_{W}\equiv {\left[P1_{\mathrm{bulk}}\right]}_{W}+{\left[P2_{\mathrm{bulk}}\right]}_{W},$$

where [P1_bulk_]_W_ = (1–*γ*)[P_bulk_]_W_, [P2_bulk_]_W_ = *γ*[P_bulk_]_W_, and *γ* is a constant (0≤*γ* ≤1). Eq. (5) is rewritten as:

(7) $$dR/dt=G{\left[P_{\mathrm{bulk}}\right]}_{W}((1–\gamma )k_{c,P1}+\gamma k_{c,P2}).$$

With respect to the SPR response (d*R*/d*t*)_exp_ experimentally determined, one can determine the concentration. When all the analytes are assumed to be the monomer [i.e., *γ*=0 in Eq. (7)], the determined concentration (as a weight concentration), *c*_WP_, is:

(8) $$c_{\mathrm{WP},\gamma =0}={\left(dR/dt\right)}_{\exp}/(Gk_{c,P1}),$$

where *c*_WP, *γ*=0_ is *c*_WP_ when *γ*=0. On the other hand, an exact concentration given by Eq. (7) is:

(9) $$c_{\mathrm{WP}}={\left[P_{\mathrm{bulk}}\right]}_{W}={\left(dR/dt\right)}_{\exp}/\{G((1–\gamma )k_{c,P1}+\gamma k_{c,P2})\},$$

The ratio between *c*_wP_ and *c*_wP__, *γ*=0_ is:

(10) $$c_{\mathrm{WP}}/c_{\mathrm{WP},\gamma =0}=k_{c,P1}/((1–\gamma )k_{c,P1}+\gamma k_{c,P2})=1/(1–\gamma +\gamma {\left(k_{c,P1}/k_{c,P2}\right)}^{–1}).$$

On the basis that the value of *k*_c_ depends on the diffusion coefficient, *D*, of the analyte [2], if the analytes are the linked dimer only [i.e., *γ*=1 in Eq. (7)] in actual, the ratio between *c*_WP_ and *c*_WP__, *γ*=0_ is:

(11) $$c_{\mathrm{WP}}/c_{\mathrm{WP},\gamma =0}=k_{c,P1}/k_{c,P2}={\left(D_{P1}/D_{P2}\right)}^{2/3}={\left(s_{P1}/s_{P2}\right)}^{2/3}{\left(M_{P1}/M_{P2}\right)}^{–2/9},$$

where *s* is (*f/f*_0_)^−1^*v*^−1/3^. *f/f*_0_ and *v* are the friction factor and the specific volume, respectively, of the analyte. When the friction factor and the specific volume of the monomer are the same as those of the linked dimer ((*f/f*_0_)_P1_=(*f/f*_0_)_P2_ and *v*_P1_=*v*_P2_), Eq. (11) is written as:

(12) $$c_{\mathrm{WP}}/c_{\mathrm{WP},\gamma =0}=k_{c,P1}/k_{c,P2}={\left(M_{P1}/M_{P2}\right)}^{–2/9}=q^{2/9},$$

where *q* is *M*_P2_/*M*_P1_. In Eq. (12), the deviation of the concentration from the actual concentration is expressed as the ratio between *k*_c,P1_ and *k*_c,P2_. The meaning of Eqs. (11), (12) are equivalent to that of the equations, Eqs. (23)–(24), in Ref. [1].

## 3 Data analysis

*k*_c,P1_/*k*_c,P2_ is expressed as:

(13) $$k_{c,P1}/k_{c,P2}=\left\{\left(dR_{P1}/dt\right)/{\left[P1_{\mathrm{bulk}}\right]}_{W}\right\}/\left\{\left(dR_{P2}/dt\right)/{\left[P2_{\mathrm{bulk}}\right]}_{W}\right\}.$$

Both (d*R*_P1_/d*t*)/[P1_bulk_]_W_ and (d*R*_P2_/d*t*)/[P2_bulk_]_W_ were experimentally available. d*R*_P1_/d*t* and d*R*_P2_/d*t* were determined by the SPR method. [P1_bulk_]_W_ and [P2_bulk_]_W_ were determined by a ultraviolet absorption measurement. The experimental data are shown in Fig. 1. The experiments gave *k*_c,P1_/*k*_c,P2_ of 1.31±0.01. The error represents the uncertainty of the value in the fitting analysis. Eq. (12) with *q*=2.86 gives *k*_c,P1_/*k*_c,P2_ of 1.26. By using the physicochemical parameters [(*f/f*_0_)_P1_=1.25 and (*f/f*_0_)_P2_=1.56 determined by the present analytical ultracentrifugation, *v*_P1_=0.730 and *v*_P2_=0.716 cm^3^/g calculated on the basis of the sequence [5], Eq. (11) gives *k*_c,P1_/*k*_c,P2_ of 1.46. We also measured the SPR of the solution of the mixture of the monomer and the linked dimer with *γ*=0.43. With respect to the relation:

(14) $$\left\{\left(dR_{P1}/dt\right)/{\left[P1_{\mathrm{bulk}}\right]}_{W}\right\}/\left\{\left(dR/dt\right)/{\left[P_{\mathrm{bulk}}\right]}_{W}\right\}=k_{c,P1}/((1–\gamma )k_{c,P1}+\gamma k_{c,P2})=1/(1–\gamma +\gamma {\left(k_{c,P1}/k_{c,P2}\right)}^{–1})=1/(1–\gamma +\gamma {\left(s_{P1}/s_{P2}\right)}^{–2/3}q^{–2/9}),$$

the experiment determined the left side of the equation to be 1.11±0.01. The right side of the equation is theoretically given as 1.10 and 1.16 with *s*_P1_/*s*_P2_=1 and *s*_P1_/*s*_P2_=1.24, respectively, the latter of which was calculated by use of the physicochemical parameters. The experimental and theoretical values were close to each other.
