# Supplementary material for: Surface plasmon resonance biosensing of the monomer and the linked dimer of the variants of protein G under mass transport limitation
Source: Data Brief. 2016 Nov 5;9:917–21. doi: 10.1016/j.dib.2016.10.029 (PMC5114526; doi:10.1016/j.dib.2016.10.029)
Supplement: Supplementary file 1 — Supplementary material [file mmc1.docx]

We wish to confirm that there are no known conflicts of interest associated with this publication.

Hiroshi Imamura

Shinya Honda

Oct 21, 2016
